# Genome-Wide Identification and Characterization of microRNAs in Developing Grains of *Zea mays* L.

**DOI:** 10.1371/journal.pone.0153168

**Published:** 2016-04-15

**Authors:** Dandan Li, Zongcai Liu, Lei Gao, Lifang Wang, Meijuan Gao, Zhujin Jiao, Huili Qiao, Jianwei Yang, Min Chen, Lunguang Yao, Renyi Liu, Yunchao Kan

**Affiliations:** 1 China-UK-NYNU-RRes Joint Libratory of insect biology, Nanyang Normal University, Nanyang, Henan, China; 2 Department of Botany and Plant Sciences, University of California, Riverside, California, United States of America; USDA-ARS, UNITED STATES

## Abstract

The development and maturation of maize kernel involves meticulous and fine gene regulation at transcriptional and post-transcriptional levels, and miRNAs play important roles during this process. Although a number of miRNAs have been identified in maize seed, the ones involved in the early development of grains and in different lines of maize have not been well studied. Here, we profiled four small RNA libraries, each constructed from groups of immature grains of *Zea mays* inbred line Chang 7–2 collected 4–6, 7–9, 12–14, and 18–23 days after pollination (DAP). A total of 40 known (containing 111 unique miRNAs) and 162 novel (containing 196 unique miRNA candidates) miRNA families were identified. For conserved and novel miRNAs with over 100 total reads, 44% had higher accumulation before the 9^th^ DAP, especially miR166 family members. 42% of miRNAs had highest accumulation during 12–14 DAP (which is the transition stage from embryogenesis to nutrient storage). Only 14% of miRNAs had higher expression 18–23 DAP. Prediction of potential targets of all miRNAs showed that 165 miRNA families had 377 target genes. For miR164 and miR166, we showed that the transcriptional levels of their target genes were significantly decreased when co-expressed with their cognate miRNA precursors *in vivo*. Further analysis shows miR159, miR164, miR166, miR171, miR390, miR399, and miR529 families have putative roles in the embryogenesis of maize grain development by participating in transcriptional regulation and morphogenesis, while miR167 and miR528 families participate in metabolism process and stress response during nutrient storage. Our study is the first to present an integrated dynamic expression pattern of miRNAs during maize kernel formation and maturation.

## Introduction

Maize kernel is one of the most important global staple foods. Elucidation of the molecular mechanism of maize kernel development will be helpful not only to the production of improved varieties of maize, but also for providing insight into seed development of other crop angiosperms. Many genes are involved in the process of kernel maturation: *ZmHOX*, *ZmOCL1*, *KN1*, *ESR*, *BETL*, and *BAP* are associated with the development of embryos and endosperms [1, 2]; *BT*, *DU*, *SH*, *SU*, and *WX* are involved in starch synthesis [3]; while *O2*, *FL2*, and *MC* participate in storage proteins formation [4–7]. Development and maturation of maize kernel involves meticulous and fine gene regulation at transcriptional and post-transcriptional levels. Although genome-wide analysis of gene expression profiles during maize kernel development has been performed, and different categories of genes have been identified at various developmental stages, the mechanisms of kernel maturation remains elusive [8–15].

Small RNAs (sRNAs) are widely recognized as important and effective regulators of gene expression in many eukaryotic organisms [16–20]. MicroRNAs (miRNAs) have attracted much attention for their various roles in post-transcriptional regulation of protein coding genes and their importance in many different pathways [21, 22], such as in the development of roots [23–26], shoots [27, 28], leaves [29–31] and flowers [32–34], as well as cell fate [35, 36]. Additionally, they were also found to play roles in responses to phytohormones [37, 38], nutrients [39–42], and environmental stresses [43–47].

Many studies have profiled maize miRNAs from different tissues as well as in plant under different stress conditions [12, 36, 45, 48–71]. So far, 172 precursors and 321 mature miRNA sequences of maize have been added to the miRNA database miRbase (Release 21; July 2014). No study has yet investigated miRNAs that are involved in very early seed development, such as the time after pollination to 10 days, or miRNAs with low abundance levels. Kang et al. studied miRNAs in immature seeds by constructing a mixed RNA library from maize inbred line B73 seeds collected 10–30 days after pollination (DAP) [50], Another recent study revealed miRNA dynamics during maize grain filling in hybrid line Zhengdan 958 [72], which originated from Zheng 58 (maternal parent) and Chang 7–2 (paternal parent), but they focused on the miRNAs expressed after the 17th DAP and miRNAs accumulating during the early stage were not included. In order to identify miRNAs that participate in the early stage of kernel development and miRNAs present in different lines of maize, we constructed four miRNA libraries from respective groups of maize inbred line Chang 7–2 immature seeds collected 4–6, 7–9, 12–14, and 18–23 DAP. Our results show a dynamic expression profile of miRNAs during maize seed development, giving us insight into the roles of miRNAs during the formation and maturation of maize kernel.

## Materials and Methods

### Small RNA library construction and RNA sequencing

Maize (*Zea mays* L.) inbred line Chang 7–2 was planted at the farmland of Henan Agricultural University (Zhengzhou, China). Immature seeds were collected 4, 5, 6, 7, 8, 9, 12, 14, 18, 20, and 23 DAP, and ground into a fine powder in liquid nitrogen and processed using RNA extraction buffer (100mM Tris-HCl, pH 8.0, 20mM EDTA, pH 8.0, 1.4M NaCl, 2.5% CTAB, 2% 2-mercaptoethanol) and an equal volume of phenol/chloroform/isoamyl alcohol (25:24:1). The aqueous fraction was subsequently extracted two times using equal volumes of phenol/chloroform/isoamyl alcohol (25:24:1) and chloroform. Total RNA was then precipitated in 5M NaCl (2.5M final concentration) and equal volumes of isopropanol overnight at –80°C. The integrity of the RNAs was verified on 1% agarose gel. Equal amounts of RNAs from each sample were then mixed into these pools: 4–6 DAP, 7–9 DAP, 12–14 DAP, and 18–23 DAP. sRNA library construction was carried out as previously described [50]. Briefly, 16- to 28-nt small RNAs were gel-purified from 15% PAGE gel, 5’ and 3’ adaptors were added, and amplified by RT-PCR using adaptor-specific primers. The PCR products were isolated and gel-purified. Sequencing was performed on the Illumina platform (BGI Inc., China).

### Bioinformatic analysis of small RNAs and miRNA identification

Analysis of small RNA data was carried out as previously described [73, 74]. Briefly, after adapter sequences and low quality reads were removed, clean small RNA reads at least 18-nt or longer were clustered into unique reads in each library. Unique reads that matched known plant structural RNAs (rRNAs, tRNAs, snRNAs, and snoRNAs) were removed from further consideration. Small RNA reads were then mapped to the maize genome sequence (release-5b+ from ftp.maizesequence.org) [75] using SOAP2 [76]. Perfect matches were required. Unique reads that had a redundancy of at least 10 copies in all libraries and were non-repetitive (mapped to less than 20 positions in the genome) were used as anchor sequences. DNA segments surrounding the anchor sequences were extracted in a stepwise fashion and were then evaluated for the potential of being a miRNA precursor based on structural characteristics and expression patterns [73, 74]. A small RNA was deemed to be a novel miRNA only if it met the strict criteria described by Ding et al. [54]. Only those candidates with a minimal folding free energy index (MFEI) > 0.85 were treated as novel miRNAs [54, 72]. Candidate mature miRNAs were classified into miRNA families based on their similarity to known plant miRNAs in miRBase (Release 21) [77] and to each other. Target genes of candidate miRNAs were predicted by using the predicted mature miRNAs as query to search annotated maize cDNAs with miRanda [78]. The alignments between miRNAs and potential targets were calculated using a position-dependent, mispair penalty system [79–81]. Each alignment was divided into two regions: a core region that included positions 2–13 from the 5’ end of the miRNA and a general region that contained other positions. In the general region, a penalty score of 1 was given to a mismatch or a single nucleotide bulge or gap, and 0.5 to a G:U pair. Penalty scores were doubled in the core region. A gene was considered a valid target if the alignment between the miRNA and target met two conditions: (1) the penalty score is 4 or less; (2) the total number of bulges and gaps is less than 2 [73, 74].

### Northern blot hybridization

Northern blot was performed as previously described [82, 83]. Approximately 4 μg of low molecular weight RNAs was separated on 15% polyacrylamide denaturing gels and then transferred electrophoretically to Hybond-N+ membranes. Membranes were UV cross-linked and baked for 2 hours at 80°C. DNA oligonucleotides complementary to miRNA sequences were end labeled with γ-^32^P-ATP using T4 polynucleotide kinase (New England Biolabs, Massachusetts, USA). Membranes were prehybridized for at least 1 hour and hybridized overnight using Perfect hybridization buffer (Sigma-Aldrich, Missouri, USA) at 38°C. Blots were washed three times (two times with 2 × SSC + 1% SDS and one time with 1 × SSC + 0.5% SDS) at 50°C. The membranes were briefly air dried and then exposed to phosphorscreen and images were acquired by scanning the films with a Typhoon phosphorimage analyzer (GE, Connecticut, USA). Blots were reprobed with an RNA probe complementary to U6 snRNA to confirm uniform loading. The Northern blot images were not quantified.

### Validation of miRNA targets by using a transient expression system

The coding sequence (CDS) or 3’- Untranslated Regions (UTR) of target genes were cloned from the cDNAs of Chang 7–2 seeds with oligo d(T)15 primers (PrimeScript^TM^ Ⅱ1st Strand cDNA Synthesis Kit, TaKaRa Dalian, China). The precursors of miRNAs were cloned from the Chang 7–2 maize genome. pCAMBIA 2300S vector with a 35S promoter was used for construction of overexpression vectors of miRNAs and their target genes.

The various constructs of target genes together with their putative miRNA precursors were transiently coexpressed in *N*. *benthamiana* according to the method by Qikun Liu and Mingda Luan [71, 84]. The *Agrobacterium tumefaciens* (Agrobacterium) strain EHA105 was transformed with the constructs pCAMBIA 2300S: miR166, pCAMBIA 2300S: Unknown CDS, pCAMBIA 2300S: Unknown CDS+3’UTR, pCAMBIA 2300S: miR164, pCAMBIA 2300S: NAM CDS, pCAMBIA 2300S: GFP. The transformed Agrobacterium suspension was infiltrated into 3-week-old *N*. *benthamiana* leaves. 48 h after transfection, leaves of *N*. *benthamiana* were washed three times with Diethy pyrocarbonate (DEPC)-treated water and dried with filter papers, RNAs were extracted using TRIzol (Thermo Fisher Scientific, MA USA). The relative expression levels of the target genes were measured with quantitative real-time PCR. *N*. *benthamiana* 18S rRNA was used as an internal control for normalization. The primers used in qRT-PCR are listed in S4 Table.

### Quantitative Real-time PCR of miRNAs and target genes

RNAs were extracted from immature maize seeds collected 6, 9, 14, and 20 DAP. Genomic DNA was digested using RNase-free DNaseⅠ(Fermentas, Ontario, Canada). cDNA synthesis and quantitative real-time PCR (qRT-PCR) of miRNAs were performed using the All-in-One™ miRNA qRT-PCR Detection Kit (GeneCopoeia, Maryland, USA). U6 was used as an internal control. For qRT-PCR of coding genes, PrimeScript^TM^ Ⅱ1st Strand cDNA Synthesis Kit (TaKaRa, Dalian, China) and SYBR Green FS Universal SYBR Green Master (Roche Applied Science, Indiana, USA) were used. PCR was carried out on the CFX96^TM^ Real-Time PCR Detection System (Bio-Rad, California, USA). The thermal cycling program consists of an initial denaturation step at 95°C for 10 min, then 40 cycles at 95°C for 15 s, 55°C for 30 s, and 72°C for 30 s. Target gene abundance in each sample was normalized according to the U6 expression (for miRNAs) or *TUBULIN* expression (for coding genes) levels using the formula ΔCt = Ct (target gene)–Ct (U6) or Ct (*TUBULIN*). The experiment was performed with three biological repeats, each with three technical repeats. The primers used for qRT-PCR are listed in S4 Table.

## Results and Discussion

### Deep sequencing of four small RNA libraries from maize seed

In order to study the roles of miRNAs involved in seed development, four sRNA libraries from four time spans of immature seeds (4–6 DAP, 7–9 DAP, 12–14 DAP, and 18–23 DAP) of maize inbred line Chang 7–2 were sequenced using Illumina 1G Sequencer. A total of 17,070,747; 17,303,264; 16,747,250; and 17,681,919 raw reads were obtained from the four libraries, respectively (S1 Table). After removing the low quality reads and adaptor sequences, the remaining clean reads from the four libraries were aligned to the maize genome. Sequences that matched perfectly to the B73 genome (B73 RefGen_v3; Release 5b+ in June, 2013) represented 66.29% to 78.38% of total reads, which indicated that the libraries were relatively intact. Then, a total of 4,041,934; 4,174,092; 3,409,610; and 3,546,660 unique reads were obtained from four libraries, respectively (S1 Table). The majority of the sRNAs were 20–24 nt long, with the 24-nt sRNA being the most abundant, followed by the 22- and 21-nt classes (S1 Fig). This result was consistent with recent reports of maize sRNAs [49–51] and similar to that of *Medicago truncatula* [85], rice [86], peanut [87], and Arabidopsis [88], which suggest that the majority of miRNAs are 24-nt in plants. Further analysis showed that approximately 0.01% of unique reads matched to miRNAs, indicating that in maize grains, miRNAs account for a proportion of ten thousandth in total RNAs. Analysis of nucleotide bias of miRNAs at each position showed that the first nucleotide of miRNAs tended to be U (S2 Fig), which is typical of most miRNAs. In addition, there was an average of 1.88%, 0.24%, 0.05%, 0.02%, and 2.29% unique reads that matched other non-coding RNAs including rRNAs, tRNAs, snRNAs, snoRNAs, and siRNAs, respectively. Protein coding RNAs consisted of 22% of the reads, and the remaining > 70% of the reads mapped to other sRNAs (Table 1).

**Table 1:** Summary of Signatures that Match Various RNAs.

Class	Unique Reads			
Non-coding RNAs	4–6 DAP	7–9 DAP	12–14 DAP	18–23 DAP
rRNA	90675 (1.31%)	112367 (1.59%)	147533 (2.61%)	116790 (2.01%)
snRNA	3003 (0.04%)	3757 (0.05%)	3667 (0.06%)	2688 (0.05%)
snoRNA	833 (0.01%)	1142 (0.02%)	1144 (0.02%)	701 (0.01%)
tRNA	8332 (0.12%)	11940 (0.17%)	21459 (0.38%)	15717 (0.27%)
miRNAs	617 (0.01%)	691 (0.01%)	648 (0.01%)	561 (0.01%)
siRNA	200634 (2.90%)	202721 (2.87%)	117761 (2.08%)	111675 (1.92%)
Protein coding RNAs				
Sense exon	403966 (5.83%)	434752 (6.15%)	390736 (6.91%)	386917 (6.65%)
Antisense exon	226615 (3.27%)	231351 (3.27%)	192670 (3.41%)	194424 (3.34%)
Sense intron	555066 (8.01%)	582015 (8.23%)	522209 (9.24%)	496060 (8.25%)
Antisense intron	272257 (3.93%)	275545 (3.90%)	225428 (3.99%)	225503 (3.87%)
Other sRNAs	5164258 (74.56%)	5217868 (73.76%)	4028157 (71.28%)	4270269 (73.36%)
Total	6926256	7074149	5651412	5821305

Among the total unique sRNA reads, 12.18% was present in all four libraries. Libraries made from seeds closer in age shared more sRNAs in common, while libraries with larger time span differences shared relatively fewer sRNAs (S3 Fig). For example, the 4–6 DAP and 7–9 DAP libraries shared 15.30% of total sRNA reads, while the 4–6 DAP and 18–23 DAP libraries shared only 12.18% (S3 Fig).

### Conservation of miRNAs in maize

To contribute to our understanding of sRNA function and conservation, candidate miRNA sequences were aligned to mature plant miRNAs and precursors. We found 111 miRNAs that could be classified into 40 known miRNA gene families (S2 Table). Because miRNA precursors are less conserved than other RNAs [89], and the minimal free energy index (MFEI) is an important criterion for distinguishing miRNAs from other sRNAs, the strict criteria described by Ding et al. [54] was used to predict the novel miRNAs. Then, a total of 196 unique miRNAs (from 162 miRNA families) were identified as novel miRNAs according to sequence identity (S3 Table). However, novel miRNAs were expressed at relatively low levels, with 6,752 Reads per million (RPM) being the highest relative abundance and 10 RPM being the lowest throughout the four libraries. Only 10% of the novel miRNAs had reads higher than 100 RPM.

### Predicted target genes of miRNAs

We predicted the targets of novel maize miRNAs through computational methods. A total of 221 and 156 predicted targets were detected from 155 novel (from 125 miRNA families) and 108 conserved (from 40 miRNA families) miRNAs, respectively.

The predicted target genes of miRNAs with total reads over 100 RPM were related to oxidoreductase activity, transcriptional regulation, transposon activity, stress response, and development. On the contrary, target genes of miRNAs with total reads less than 100 RPM were related to various biological processes (S2 and S3 Tables).

### Validation of target genes of miRNAs

**Fig 1:**
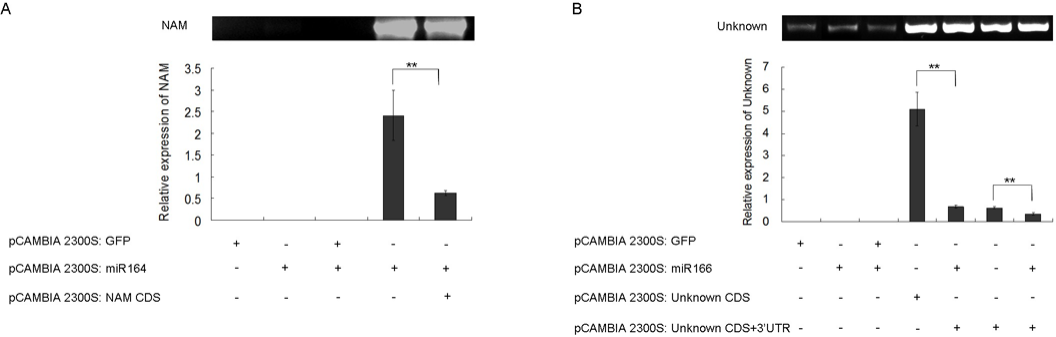
Coexpression of miRNA precursors and their target genes in a transient expression system in *N*. *benthamiana* cells. (A) Relative expression levels of NAM gene coexpressed with miR164 precursor in *N*. *benthamiana*. (B) Relative expression levels of unknown gene coexpressed with miR166 precursor in *N*. *benthamiana*. Target genes were also coexpressed with an unrelated *GFP* construct as a control. Tobacco 18s rRNA was used as an internal control for normalization.

To confirm the interaction between target genes and their cognate miRNAs in vivo, two miRNAs, miR166 and miR164 with higher expression in the early development of maize kernel were chosen. Given the miR164 target sites was located in the coding sequence (CDS) of No apical meristem (*NAM*) gene, the overexpression vectors of *NAM* CDS as well as the precursor of miR164 were constructed and subsequently coinoculated into *N*. *benthamiana* leaves. Results showed that the relative expression levels of the *NAM* transcripts decreased significantly when the precursor of miR164 was expressed (Fig 1A), which demonstrated that miR164 can reduce the abundance of its predicted target gene, probably through mediating the degradation of the *NAM* transcripts.

The target site of miR166 was located in the 3’-UTR of an uncharacterized gene (Unknown). The interaction of miR-166 with this unknown gene was also investigated by the same method as with miR164. Detected expression levels of the unknown gene decreased significantly when the precursor of miR166 was expressed (Fig 1B), suggesting that miR166 can reduce the expression level of this unknown gene through interaction with the 3’-UTR region.

### Expression pattern of miRNAs showed that different miRNA families might play dominant roles at distinct developmental stages in maize seed

**Fig 2:**
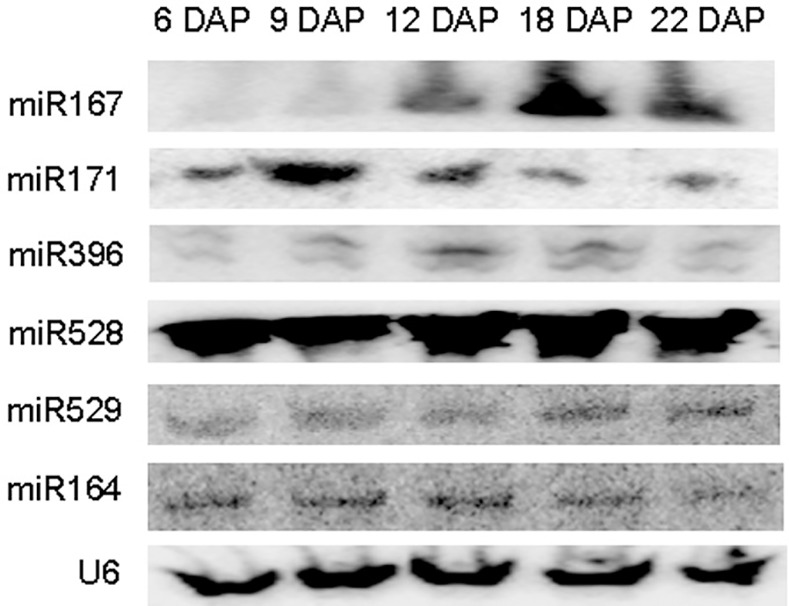
Northern blot analysis of selected maize miRNAs. **Maize** *U6* RNA was used as an internal control. DAP: days after pollination.

**Fig 3:**
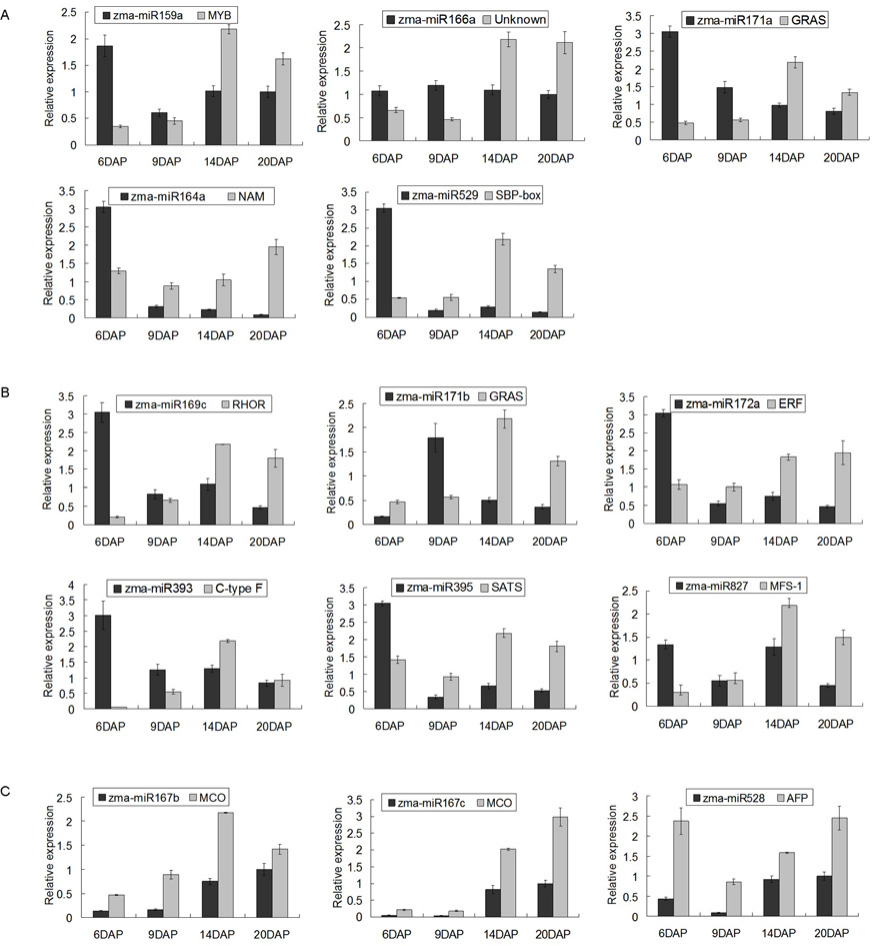
Relative expression levels of 14 miRNAs and their target genes at four time points during maize seed development. *U6* and *TUBULIN* were used as internal controls for miRNAs and target genes, respectively. DAP: days after pollination. Maize miRNAs are indicated by dark gray bars; target genes are indicated by light gray bars.

Maize grains take around 35 days to fully mature, and 10–15 DAP is the critical transition stage of maize seed development. Before the 15^th^ DAP, embryo development is focused on formation of the tissues and organs, while storage of nutrients in the kernel begin after the 10^th^ DAP [90]. From the 15^th^ DAP onward, the accumulation of storage nutrients is accelerated [50]. We profiled the miRNA abundance from four developmental time spans of maize immature seeds (4–6 DAP, 7–9 DAP, 12–14 DAP, and 18–23 DAP). We chose 21 miRNAs with significant expression variations across the four developmental time spans for further analysis. The expression of six miRNAs were verified by Northern Blot and were consistent with the sequencing results (Fig 2), while the others had weak or no hybridization signals most likely due to low abundance as seen in the low sequencing reads. In addition, the expression pattern of 14 miRNAs at four time points (6 DAP, 9 DAP, 14 DAP and 20 DAP) during maize seed development were also verified by qRT-PCR. With the exception of miR166, whose expression did not vary across the four stages, and miR169, miR172, miR393, and miR395, which all had relatively more expression at the 6^th^ DAP, the others were consistent with the sequencing data (Fig 3). The RNA libraries prepared for sequenceing were generated from mixed samples of seeds form different days during development, but here we used single DAP samples to detect the expression of miRNAs, which might explain the discrepancies.

Since miRNAs with higher expression levels suggest their fixed regulatory roles during the miRNA evolution, we chose conserved miRNA family members and miRNAs with total reads over 100 RPM for further analysis, and then 109 miRNAs (44 families) were filtered. According to the criteria described by Jin et al. [72], the expression pattern of miRNAs were categorized in six groups (Table 2). Although the study in Zhangdan 958 revealed that a large number of miRNAs accumulated more at 25 DAP [72], we found that only 16% of the miRNAs had more accumulation 18–23 DAP, and the remaining 84% of miRNAs had higher accumulation before the 14^th^ DAP.

**Table 2:** Expression Pattern of Known miRNAs in Maize Grain Development.

Name	4–6 DAP RPM	7–9 DAP RPM	12–14 DAP RPM	18–23 DAP RPM	Changing Pattern
zma-MIR156	11	15	26	115	a
zma-MIR159a	2520	1851	832	339	b
zma-MIR159b	2520	1851	832	339	b
zma-MIR159c	2520	1851	832	339	b
zma-MIR159d	2520	1851	832	339	b
zma-MIR159e	2520	1851	832	339	b
zma-MIR160a	5	194	85	64	d
zma-MIR160b	5	194	85	64	d
zma-MIR160c	5	194	85	64	d
zma-MIR160d	5	194	85	64	d
zma-MIR160e	5	194	85	64	d
zma-MIR164a	16299	10208	3865	1317	b
zma-MIR164b	16299	10208	3865	1317	b
zma-MIR164c	16299	10208	3865	1317	b
zma-MIR164d	16299	10208	3865	1317	b
zma-MIR164e	342	206	63	23	b
zma-MIR164f	342	206	63	23	b
zma-MIR164g	59	16	6	3	b
zma-MIR166a	227674	132545	80152	62546	b
zma-MIR166b	227674	132545	80152	62546	b
zma-MIR166c	227674	132545	80152	62546	b
zma-MIR166d	227674	132545	80152	62546	b
zma-MIR166e	227674	132545	80152	62546	b
zma-MIR166f	35948	28525	9378	4442	b
zma-MIR167a	4193	12187	82693	79897	e
zma-MIR167b	4193	12187	82693	79897	e
zma-MIR167c	4193	12187	82693	79897	e
zma-MIR167d	3077	3609	5370	14089	a
zma-MIR167e	3077	3609	5370	14089	a
zma-MIR167f	3077	3609	5370	14089	a
zma-MIR167g	3077	3609	5370	14089	a
zma-MIR167h	45	971	59200	105477	a
zma-MIR167i	45	971	59200	105477	a
zma-MIR167j	45	971	59200	105477	a
zma-MIR169a	36	72	228	73	e
zma-MIR169b	44	52	52	21	d
zma-MIR169c	138	223	88	40	d
zma-MIR169d	2	13	37	17	e
zma-MIR171a	3124	1695	944	413	b
zma-MIR171b	3124	1695	944	413	b
zma-MIR171c	336	512	3422	2362	e
zma-MIR171d	336	512	3422	2362	e
zma-MIR171e	336	512	3422	2362	e
zma-MIR171f	336	512	3422	2362	e
zma-MIR171g	336	512	3422	2362	e
zma-MIR171h	336	512	3422	2362	e
zma-MIR171*a	37	47	10	7	d
zma-MIR171*b	5	2	4	1	c
zma-MIR171*c	37	47	10	7	d
zma-MIR172a	117	253	548	211	e
zma-MIR172b	117	253	548	211	e
zma-MIR172c	117	253	548	211	e
zma-MIR390a	320	169	73	76	c
zma-MIR390b	320	169	73	76	c
zma-MIR393a	3	58	75	72	e
zma-MIR393b	3	58	75	72	e
zma-MIR393c	3	58	75	72	e
zma-MIR394a	2	64	42	47	c
zma-MIR394b	2	64	42	47	c
zma-MIR395a	18	28	50	23	e
zma-MIR395b	18	28	50	23	e
zma-MIR395c	18	28	50	23	e
zma-MIR395d	18	28	50	23	e
zma-MIR395e	18	28	50	23	e
zma-MIR395f	18	28	50	23	e
zma-MIR395g	18	28	50	23	e
zma-MIR395h	18	28	50	23	e
zma-MIR395i	18	28	50	23	e
zma-MIR395j	18	28	50	23	e
zma-MIR395k	18	28	50	23	e
zma-MIR396a	3632	5092	3080	1782	d
zma-MIR396b	3632	5092	3080	1782	d
zma-MIR396c	402	438	389	248	d
zma-MIR396d	402	438	389	248	d
zma-MIR396*	20	27	24	16	d
zma-MIR399a	42	39	20	9	b
zma-MIR399b	42	39	20	9	b
zma-MIR399c	42	39	20	9	b
zma-MIR399d	12	12	16	2	d
zma-MIR398	10	7	23	51	f
zma-MIR528a	13381	3265	16847	69688	f
zma-MIR528b	13381	3265	16847	69688	f
zma-MIR529	281	184	5	7	c
zma-MIR827	3898	5224	8686	1937	e
zma-MIR1432	25	8	12	16	c

a, miRNAs whose abundance increased linearly from 4 to 23 DAP

b, miRNAs whose abundance decreased linearly from 4 to 23 DAP

c, miRNAs with highest expression at 4–6 DAP

d, miRNAs with highest expression at 7–9 DAP

e, miRNAs with highest expression at 12–14 DAP

f, miRNAs with highest expression at 18–23 DAP

* denotes the miRNAs originates from the other strand of the miRNA:miRNA* duplex.

Group e, the miRNAs with the highest expression at 12–14 DAP, formed the largest group with a percentage of 42%, but their total reads were relatively lower. This group includes 11 conserved and 7 novel miRNA families, such as the miR169, miR171, miR172, miR393, miR395 and miR827 families. Target genes of miR169, miR171 and miR172 families are transcription factors; and target genes of miR393, miR395 and miR827 families encode transporter related proteins (S2 Table), which are involved in signal transductions. The expression pattern of all miRNAs, with the exception of the miR827 family, and their target genes were negatively correlated (Fig 3B). The accumulation of these miRNAs at 12–14 DAP indicate that they might play roles in transcriptional regulation and signal transduction during the transition from embryogenesis to nutrient storage of maize kernel.

We found that miR159, miR164, miR166, miR171, miR390, miR393, and miR529 families all accumulated to high levels during the very early stage of development (4–6 DAP), especially miR166. Kang et al., also found that miR166 was highly expressed in developing seeds [50], but they did not reveal the exact stage. Here, we found that miR166 had highest expression at the very early stage of seed maturity in Chang 7–2. This differs from Zhengdan 958, the offspring of Zheng 58 (maternal parent) and Chang 7–2 (paternal parent), from which miR166 had higher accumulation after 25 DAP [72].

Target genes of miR159, miR166, miR171, and miR529 are transcription factors (S2 Table). The expression profiles of miRNAs and their target genes at four time points of maize kernels development were detected by qRT-PCR. Results show that miR159, miR166, miR171, and miR529 had relatively higher expression before the 10^th^ DAP, which corresponded to the deep-sequencing data, while their target genes had opposite expression trends, with more accumulation after the 14^th^ DAP (Fig 3A). miR166 was predicted to target basic-leucine zipper (bZIP) genes in maize [24], which could regulate many processes like seed maturation, stress signaling, and flowering timing [50]. In Arabidopsis, miR166/165 targets class III homeodomain leucine zipper (HD-ZIP III) transcription factors, which determine the fate of the shoot apical meristem (SAM), AGO10 acts as a decoy and sequesters miR166/165 from AGO1, thus preventing the silencing of the HD-ZIP III genes and resulting in defective SAM [28]. Here we found that the target gene of miR166 was an uncharacterized gene. The accumulation of miR166 in the early stage and the corresponding down-regulated expression of its target gene seen in our results indicate that it might play roles in transcriptional regulation in the embryogenesis of maize kernel.

Target genes of miR164 encode No apical meristem (NAM) proteins (S2 Table), The *NAM* family genes encode transcription factors that play critical roles in boundary formation and lateral organ separation, which is important for proper leaf and flower patterning [91]. The function of miR164 in posttranscriptional regulation of *NAM* genes is conserved in many plants [92–95]. Our results showed that miR164 has higher expression during early development of maize seed while *NAM* expression is low, and the *NAM* gene has higher expression during the latter stages (Fig 3A). The early stage accumulation of miR164 indicates that it might play roles in the embryogenesis of maize kernel through silencing *NAM* genes.

Conserved miRNAs that accumulated more in the late developmental stage of maize seed include miR156, miR167, miR398, and miR528 families. Expression of miR156 increased linearly and was the highest at 18–23 DAP, which was consistent with the results seen in Zhengdan 958 [72], as well as in rice [96], wheat [97] and barley [98]. The expression pattern of the miR167 family varied; some variants had highest expression level at 12–14 DAP, while others increased linearly and had highest accumulation at 18–23 DAP. Variants that originated from different regions on the same chromosome had differential expression patterns, similar to the results seen in Zhengdan 958 [72]. Further analysis showed that the target gene of miR167 encodes a protein of monocopper oxidase (MCO) protein (S2 Table). This was different from studies which predicated the target genes encoded auxin response factors (ARF) [45, 99, 100]. The discrepancy may be attributed to different criteria used for target prediction; we had used more strict criteria that eliminates mismatches, bulges, or gaps between miRNAs and their target genes. Nucleotides 1–21 of miR167 are fully complementary to the cDNA of *MCO*, but nucleotides 3–20 of miR167 are only nearly complementary to the cDNA of *ARF* with one mismatch. In the roots and developing ears of maize, miR167 exhibited a reverse expression pattern to *ARF* [45, 48]. Here we found that the expression pattern of miR167c was consistent with the target gene *MCO* during maize seed development (Fig 3C). The commonly known role of miRNAs include is regulating gene expression by repressing translation or directing sequence-specific degradation of complementary mRNAs. But some miRNAs were found to induce gene expression through complementarity to the promoter sequence of target genes [101]. Further analysis showed that miR167 has no sequence complementarity to the promoter of *MCO* genes. Whether *MCO* gene is a *bona fide* target of miR167, and if so, by which mechanism miR167 regulates *MCO* remains to be eluciated. Liu et al. found that from 15–25 DAP, the majority (60%) of differentially expressed genes were related to metabolism, and many of them were up-regulated [90]. Our results show that miR167 has higher accumulation during the stage of nutrient storage, indicating that miR167 might be involved in the metabolism regulation of seed maturity by regulating the expression of the *MCO* gene.

Another miRNA family which showed highest accumulation in the late stage of maize kernel development was miR528, with highest expression at 18–23 DAP, which was consistent with the results of its filial line Zhengdan 958 [72]. Recent studies showed that miR528 is significantly repressed during low nitrate conditions in maize roots and shoots [48], while in *Triticum*. *dicoccoides*, miR528 was down-regulated in leaves during drought stress [102], pointing to the role of miR528 in stress response. The candidate target gene of of miR528 encodes antifreeze protein (AFP), and the expressions of miR528 and *AFP* gene both increased in latter stages during maize seed development (Fig 3C). Further analysis revealed that miR528 has no sequence complementarity to the promoter region of *AFP* genes, which indicates that the expression of *AFP* gene might be influenced by other factors in addition to miR528. The accumulation of miR528 in the late development of maize kernel suggests that it might participate in the control of stress responses during the process of nutrient storage by regulating the expression of *AFP* gene.

**Fig 4:**
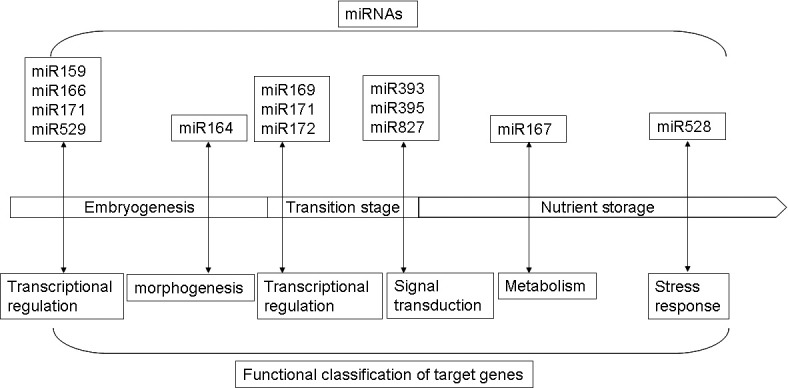
A model of putative roles of miRNAs and their target genes involved in maize grain development.

In conclusion, conserved miRNA families that accumulate to higher levels during the early stage of maize seed development such as miR159, miR164, and miR166 might play roles by participating in transcriptional regulation and morphogenesis. Conserved miRNA families with higher expression at the transitional stage such as miR169, miR171, and miR393 might play roles in transcription regulation and signal transduction. Finally, the miRNA families with higher expression levels in the latter stage of nutrient storage such as miR167 and miR528 might participate in metabolism and stress response (Fig 4).

## Conclusions

We studied the expression profile of miRNAs during maize seed development using sRNA deep sequencing, Northern blot and qRT-PCR analysis. We identified 111 conserved miRNAs (from 40 miRNA families) and 196 novel miRNAs (from 162 miRNA families) in the immature grains of maize. 44% of the miRNAs had higher accumulation before the 10^th^ DAP, 42% had highest expression at 12–14 DAP, and only 14% miRNAs had higher abundance at 18–23 DAP. A total of 377 target genes were predicted from 165 miRNA families, the interaction between miR164, miR166 and their target genes were confirmed in vivo. miR159, miR164, miR166, miR171, miR390, miR399, and miR529 families might play roles in the embryogenesis of maize grain by participating in transcriptional regulation and morphogenesis, while miR167 and miR528 families might play roles in the process of nutrient storage by participating in the metabolism process and stress response. Our study is the first to reveal an intact dynamic expression profile of miRNAs during maize seed development.

## Supporting Information

S1 Fig Length distribution of small RNAs from four libraries. (TIF)

S2 Fig Nucleotide bias at each position in miRNAs among the four small RNA libraries. A, B, C, and D represented the libraries made from seeds cellected 4–6 DAP, 7–9 DAP, 12–14 DAP and 18–23 DAP, respectively.
(TIF)

S3 Fig Common and specific unique reads among the four small RNA libraries. 5d: 4–6 DAP, 7d: 7–9 DAP, 12d: 12–14 DAP, 18d: 18–23 DAP.
(TIF)

S1 Table Summary of high-throughput small RNA sequencing. (XLS)

S2 Table Information and target genes of conserved miRNAs in the developing seeds of maize. (XLS)

S3 Table Information and target genes of novel miRNAs in the developing seeds of maize. (XLS)

S4 Table Primer sets used in quantitative real-time PCR of miRNAs and target genes. (XLS)
