# Supplementary figures and images for: Genome-Wide Identification and Characterization of microRNAs in Developing Grains of Zea mays L
Source: PLoS One. 2016 Apr 15;11(4):e0153168. doi: 10.1371/journal.pone.0153168 (PMC4833412; doi:10.1371/journal.pone.0153168)

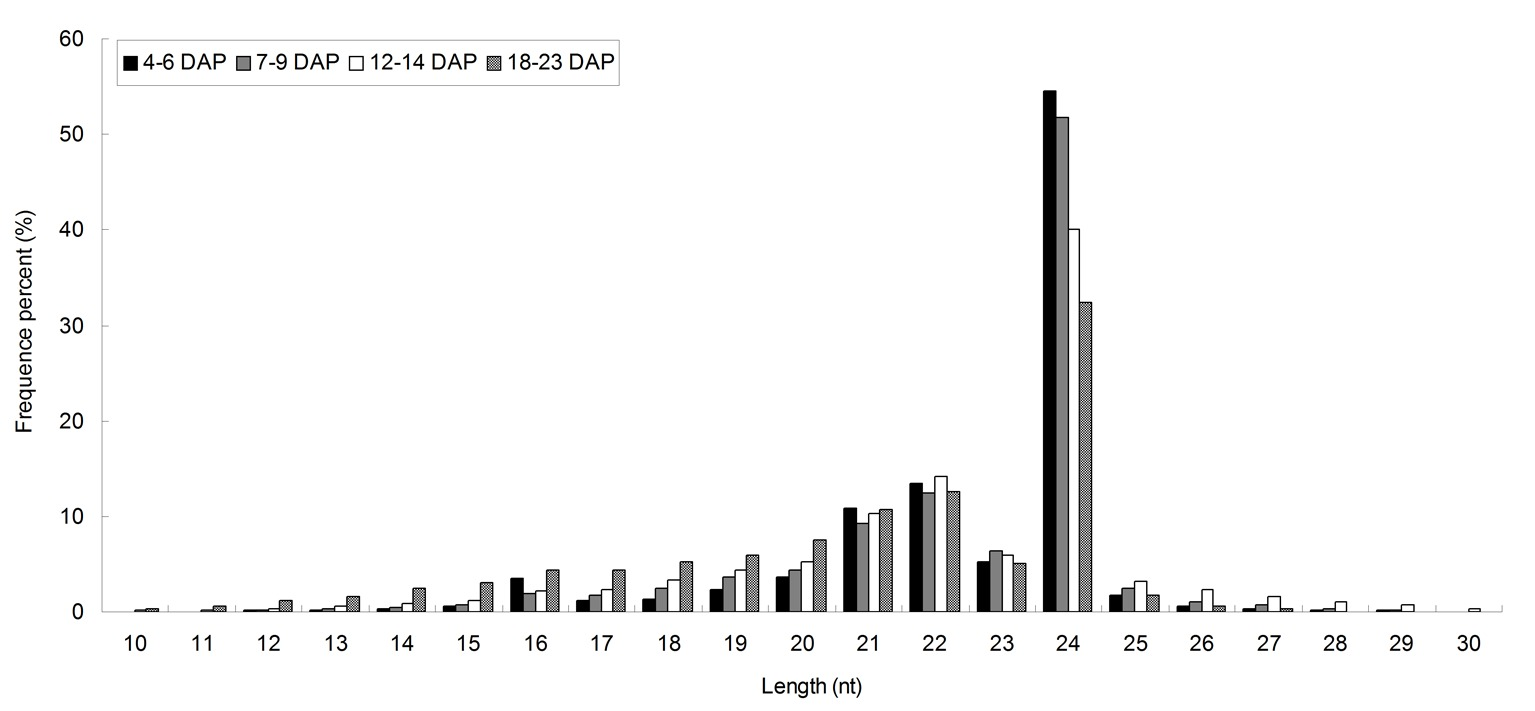

Supplement: S1 Fig — (TIF) [file pone.0153168.s001.tif]

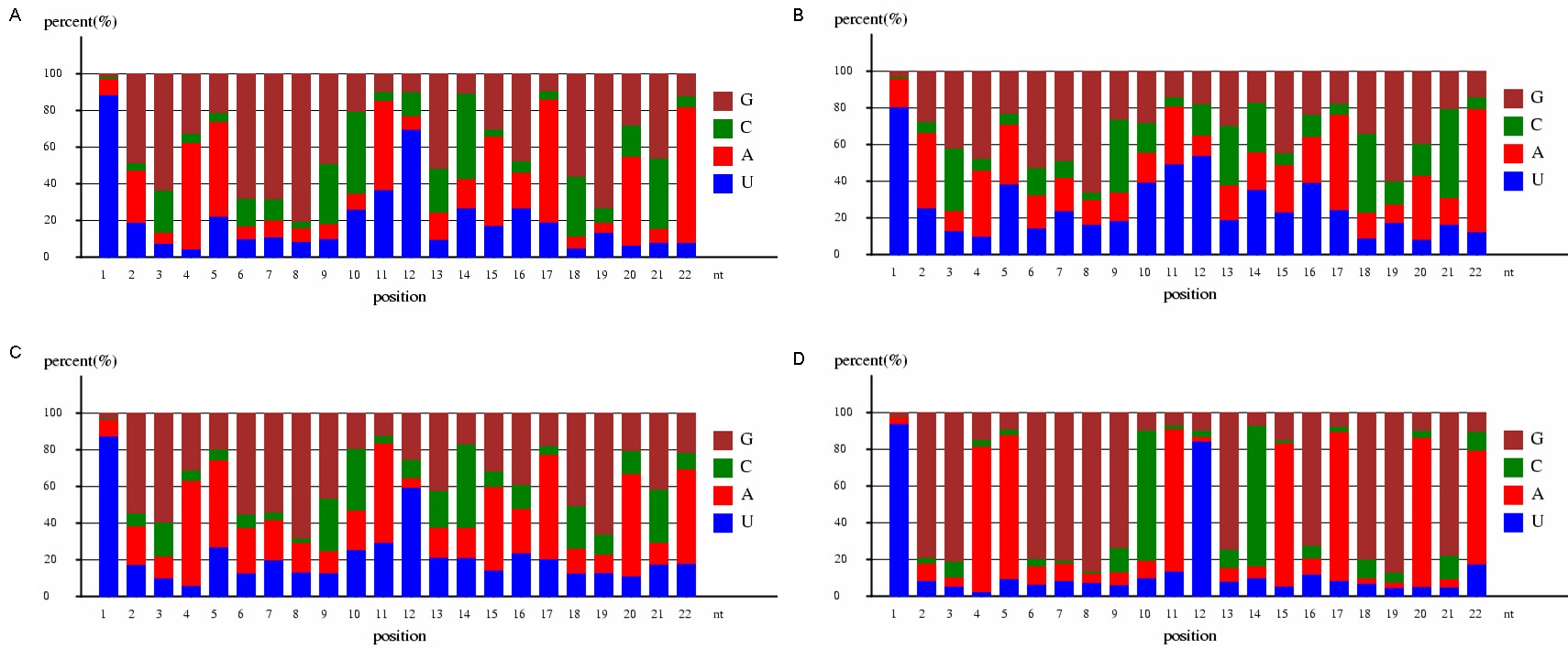

Supplement: S2 Fig — A, B, C, and D represented the libraries made from seeds cellected 4–6 DAP, 7–9 DAP, 12–14 DAP and 18–23 DAP, respectively. (TIF) [file pone.0153168.s002.tif]

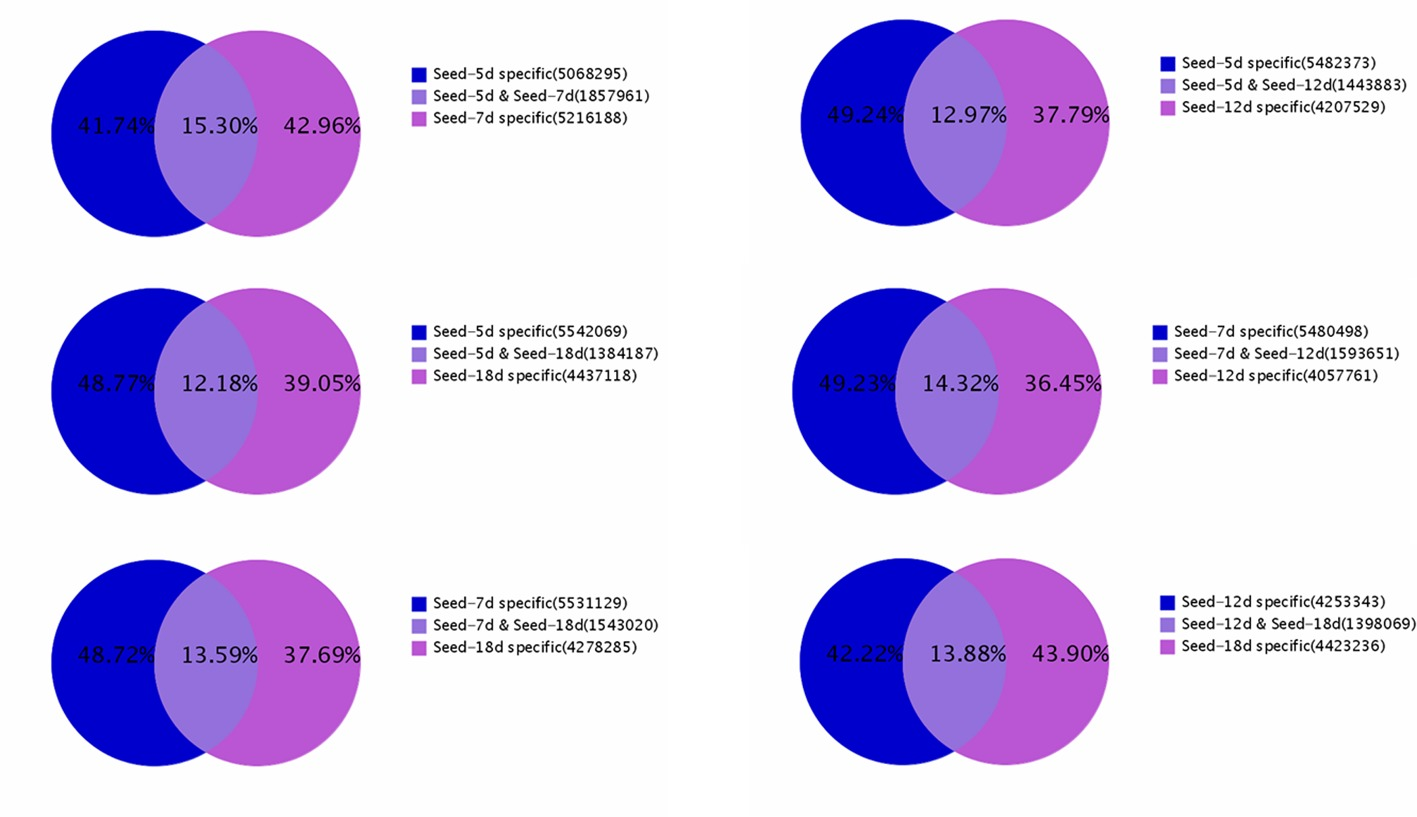

Supplement: S3 Fig — 5d: 4–6 DAP, 7d: 7–9 DAP, 12d: 12–14 DAP, 18d: 18–23 DAP. (TIF) [file pone.0153168.s003.tif]
